# Effectiveness and antimicrobial susceptibility profiles during primary antimicrobial prophylaxis for pediatric acute myeloid leukemia

**DOI:** 10.1038/s41598-021-00725-5

**Published:** 2021-10-27

**Authors:** Ting-Chi Yeh, Jen-Yin Hou, Ting-Huan Huang, Chien-Hung Lu, Fang-Ju Sun, Hsiu-Mei Huang, Hsi-Che Liu

**Affiliations:** 1 Division of Pediatric Hematology-Oncology, Department of Pediatrics, Mackay Children’s Hospital and Mackay Medical College, 92, Section 2, Chung-San North Road, Taipei, 104 Taiwan; 2 Division of Pediatric Hematology-Oncology, Department of Pediatrics, Hsinchu Mackay Memorial Hospital, Hsinchu, Taiwan; 3 Division of Clinical Pharmacy, Department of Pharmacy, MacKay Memorial Hospital, Taipei, Taiwan; 4 Department of Medical Research, MacKay Memorial Hospital, Taipei, Taiwan; 5 School of Nursing, National Taipei University of Nursing and Health, Science, Taipei, Taiwan

**Keywords:** Cancer, Microbiology, Oncology

## Abstract

Limited data are available on antimicrobials exposure and microbiology evolution in pediatric acute myeloid leukemia (AML) patients underwent antimicrobials prophylaxis. To assess the effectiveness of antimicrobials prophylaxis, antibiotic susceptibilities of bacteria, and exposure of antimicrobials during intensive chemotherapy for AML patients, 90 consecutive de novo AML patients aged 0–18 years between January 1, 1997 and March 31, 2018 were enrolled. Vancomycin, ciprofloxacin and voriconazole prophylaxis was administered from January 1, 2010. During the preprophylaxis period, January 1997 to December 2009, 62 patients experienced a total of 87 episodes of bloodstream infection (BSI) and 17 episodes of invasive fungal infection (IFI) among 502 courses of chemotherapy. In contrast, 16 episodes of BSI occurred and no IFIs were reported to occur in 28 patients who received 247 courses of chemotherapy in the prophylaxis period. Patients who received antimicrobial prophylaxis had a significant reduction of BSI, IFI, and febrile neutropenia in comparison with patients without prophylaxis. Exposure to amikacin, carbapenem, amphotericin B was reduced in the prophylaxis period. Imipenem susceptibility of *Enterobacter cloacae* as well as vancomycin susceptibility of *Enterococcus* species were reduced in the prophylaxis period. At the time of the last follow up, patients with prophylaxis had a better subsequent 5-year overall survival rate than those without prophylaxis. Prophylactic antimicrobials administration in children with AML who undergo chemotherapy can significantly reduce the rates of life-threatening infection, exposure to antimicrobials, and might result in a better outcome.

## Introduction

Outcomes for pediatric acute myeloid leukemia (AML) patients have improved remarkably with a 70% improvement rate over the past decades^1^. Risk-directed treatment strategy, adjusted chemotherapy dosing or timing and intensification of therapy for disease eradication, better supportive-care measures to reduce early death and treatment-related mortality, and efficacy of salvage therapy contributed to this improvement^2–5^. Approximately 60% of children with AML who underwent chemotherapy experienced life-threatening infections with a reported cumulative infection-related mortality (IRM) of 6 to 11%^6,7^. To reduce the IRM, an approach of prophylaxis with antibiotics and antifungal agents in pediatric AML patients was reasonable and has been reported^5,8–10^. The use of prophylactic antibiotics in adult AML patients with afebrile neutropenia following chemotherapy is supported by meta-analyses of randomized trials demonstrating reduced the occurrence of clinically documented infections and risk of infection-related death^11^. Data describing the effectiveness of the use of antimicrobial prophylaxis in pediatric AML patients with afebrile neutropenia are limited^5,8–10^. Furthermore, there has been no detailed report on the comparison of antimicrobial exposure and changes in bacterial susceptibility during antimicrobial prophylaxis in pediatric AML patients. The primary objective of the current study was to investigate the effectiveness of prophylaxis with antibiotic and antifungal agents in the prevention of bloodstream infection (BSI), invasive fungal infection (IFI), febrile neutropenia (FN), and outcome during intensive chemotherapy for AML patients. A secondary aim was to assess antimicrobials susceptibilities of the major gram-negative bacteria (GNB), gram-positive bacteria (GPB), and *Candida species*, and the exposure of antimicrobials during the study period.

## Materials and methods

### Patients and protocols

This was a single-center, observational cohort study of patients with newly diagnosed AML at Mackay Children’s Hospital in Taipei, Taiwan. From January 1, 1997 through March 31, 2018, 90 consecutive children with AML who were younger than 18 years old and who did not have Down syndrome, acute promyelocytic leukemia, or therapy-related AML were enrolled in this study. The Institutional Review Board of MacKay Memorial Hospital approved the study and all of the participants or their guardians provided written, informed consent of AML treatment, in accordance with the Declaration of Helsinki. Patients with newly diagnosed AML were treated with the Taiwan Pediatric Oncology Group (TPOG)-AML-97A protocol (activated in January 1997) consisting of induction chemotherapy, post-remission high-dose (HD) and modest-dose (MD) chemotherapy as previously described (see Supplemental Figure S1 online)^12,13^. Minimal residual disease (MRD) measurement for AML with *RUNX1-RUNX1T1*, *CBFB-MYH11*, *MLLT3-KMT2A* fusion was started in 2013.

From January 1, 2010 to March 31, 2018, prophylaxis with antibiotic and antifungal agents was administered to afebrile neutropenic patients with AML who received induction as well as post-remission HD and MD chemotherapy. Each chemotherapy course was analyzed separately for occurrence of BSI, probable and proven IFIs, FN during the preprophylaxis period (1997–2009) and prophylaxis period (January 1, 2010–March 31, 2018). Microbiological organisms of BSI and IFI, treatment outcome, antibiotic susceptibilities of the major GNB and GPB at the study institution and exposure of antimicrobials for any causes during AML-97A chemotherapy were recorded to compare the preprophylaxis and the prophylaxis periods. The outcomes of the AML were censored on September 30, 2020, with a duration after completion of chemotherapy of more than 2 years and 6 months.

### Antimicrobial prophylaxis protocols

Oral ciprofloxacin (at a dose of 300 mg/m^2^ every 12 h) and voriconazole (7 mg/kg every 12 h, maximum dose of 200 mg) were administered once an afebrile patient’s absolute neutrophil count (ANC) ≤ 0.5 × 10^9^/L was reached and expected to last ≥ 7 days during chemotherapy. The incidence of GPB in children with AML treated at study institution from January 1997 to December 2012 was 5%, of which two patients resulted in death, so we started to initiated Vancomycin (400 mg/m^2^ administered intravenously every 12 h) in patients at the onset of neutropenia since January, 2013. Therapeutic drug monitoring (TDM) of voriconazole was performed to ensure efficacy and limit toxicity in patients receiving 7 days of voriconazole prophylaxis (1–5 mcg/mL as targeted trough level)^14^. TDM of vancomycin was not routinely monitored while its toxicities and tolerance were recorded. Prophylaxis with antibiotic and antifungal agents was discontinued once the ANC recovered to > 0.1 × 10^9^/L post nadir. All patients were placed on *Pneumocystis jirovecii* prophylaxis with trimethoprim–sulfamethoxazole.

### Management of febrile neutropenia

At the study institution, management strategies for a patient with febrile neutropenia included 2 blood cultures for bacteria obtained via the central venous catheter (if present) and a peripheral vein. Cefuroxime and amikacin were used as empirical antibiotics during two time periods of study. Prophylactic vancomycin and ciprofloxacin were withdrawn while voriconazole prophylaxis continued once patients experienced febrile neutropenia. These guidelines did not change during the study period.

### Definitions of infectious episodes

The National Cancer Institute’s Common Terminology Criteria for Adverse Events (version 5.0)^15^ was applied to define all infection events in which ≥ grade 3 were recorded. FN was defined as a single core body temperature ≥ 38.3 °C or ≥ 38.0 °C persisting at least one hour in context of neutropenia. BSI was defined as one recognized pathogen that was isolated from ≥ 1 blood culture without relation to an infection at another site, and clinical signs of systemic infection^16^. If one common commensal organism (e.g., methicillin-resistant coagulase-negative staphylococci) was isolated from 2 blood cultures among those patients with ANC ≤ 0.5 × 10^9^/L, it was recorded as a true pathogen of BSI. All BSI in children at any time who underwent AML-97A chemotherapy was reported. IFIs, including yeasts and molds, were graded as proven, probable, or possible in accordance with the European Organization for the Research and Treatment of Cancer/Mycoses Study Group criteria^17^. Only proven and probable IFI were recorded for analysis in the current study.

### Assessment of the exposure to antimicrobials

Specific antimicrobial days were calculated for each patient as the simple proportion of cumulative chemotherapy days on which a specific antimicrobial was administered (excluding *Pneumocystis jirovecii* pneumonia prophylaxis). Each antimicrobial exposure was calculated and compared between the preprophylaxis and prophylaxis periods. Antibiotics were grouped as follows: cephalothin or cefuroxime, ceftazidime, amikacin, piperacillin/tazobactam, carbapenem, and teicoplanin. Antifungal agents were grouped as follows: conventional or liposomal amphotericin B, caspofungin, and triazoles including fluconazole, itraconazole, and posaconazole.

### Assessment of antimicrobials susceptibility

At the study institution, bacterial antibiotic susceptibility monitoring and recording have started since 2002 and antifungal susceptibility of *Candida species* has been available since 2017. The antibiotics susceptibility for the most common 6 GNB including *Escherichia coli* (*E. coli*), *Klebsiella pneumoniae* (*K. pneumoniae*), *Enterobacter cloacae* (*E. cloacae*), *Pseudomonas aeruginosa* (*P. aeruginosa*), *Citrobacter freundii* (*C. freundii*), *Acinetobacter baumannii* (*A. baumannii*) and 3 GPB including *Staphylococcus aureus* (*S. aureus*), *Enterococcus* species (*Enterococcus* spp.), Coagulase-negative Staphylococci (CoNS) in pediatric AML patients from 2002 to 2019 were recorded. The results of antibiotic susceptibility for every bacterium were compared individually between the preprophylaxis and prophylaxis periods. We focused on the change in antibiotic susceptibility of the bacteria during the prophylaxis period.

### Statistical analysis

Comparisons of developing BSIs, IFIs, and FN presented as the proportion of chemotherapy courses, clinical and genetic features, and mortality from infections between the 2 periods were estimated using the chisquare test. Hierarchical linear modeling (HLM) was used to adjust covariates including age, duration of neutropenia, oral mucositis, and parenteral nutrition therapy for potential confounding influences of the occurrence of BSIs, IFIs, and FN during preprophylaxis and prophylaxis periods. The Kruskal–Wallis test was used to compare course and cumulative days of chemotherapy. The Mann–Whitney U test was used to compare the antimicrobials exposure and antibiotic susceptibility between the 2 periods. Kaplan–Meier estimates were used to plot overall survival (OS), event-free survival (EFS), and to graph the time to first event for FN, BSI, or IFI between the preprophylaxis and prophylaxis periods and were compared using the log-rank test. A P value < 0.05 was considered to be statistically significant. SPSS statistical software (version 21; SPSS Inc, Chicago, Ill) was used to perform the statistical analyses.

## Results

Presenting features of the 90 children were shown in Table 1. Patient’s flow chart during the study was shown in Supplemental Figure S2.

**Table 1:** Presenting characteristics.

Characteristics		Preprophylaxis (N = 62, %)	Prophylaxis (N = 28, %)	*p*
Gender	Male	33 (53)	16 (57)	0.821
	Female	29 (47)	12 (43)	
Age, years	< 10	43 (69)	15 (54)	0.162
	≥ 10	19 (31)	13 (46)	
Leukocyte counts	< 100 × 10^9^/L	50 (81)	23 (82)	1.000
	≥ 100 × 10^9^/L	12 (19)	5 (18)	
FAB subtype	M0	3 (5)	0	0.363
	M1	5 (8)	1 (4)	
	M2	18 (29)	13 (46)	
	M4	19 (31)	4 (14)	
	M5	8 (13)	6 (21)	
	M6	2 (3)	1 (4)	
	M7	7 (11)	3 (11)	
Karyotype	t(8;21)	10 (16)	9 (32)	0.188
	inv(16)	5 (8)	1 (4)	
	11q23/*KMT2A*	12 (19)	4 (14)	
	t(1;22)	0	1 (4)	
	Complex	3 (5)	3 (11)	
	Others	32 (52)	10 (36)	
Number of patient with BSI		48 (77)	10 (35)	< 0.001
Number of patient with IFI		17 (27)	0 (0)	0.002
Number of chemotherapy courses per patient		Median: 9 (range, 1–11)	Median: 9 (range, 5–14)	0.09
Number of chemotherapy days per patient		Median: 241 (range, 6–352)	Median : 235 (range, 127–546)	0.459
Number of prophylactic antimicrobial days	Vancomycin		Median: 71 (24–191)	
	Ciprofloxacin		Median: 74 (3–204)	
	Voriconazole		Median: 84 (3–174)	

BSI, bloodstream bacterial infection; FAB, French-American-British; IFI, invasive fungal infection.

### Effectiveness of antimicrobials prophyalxis

**Figure 1:**
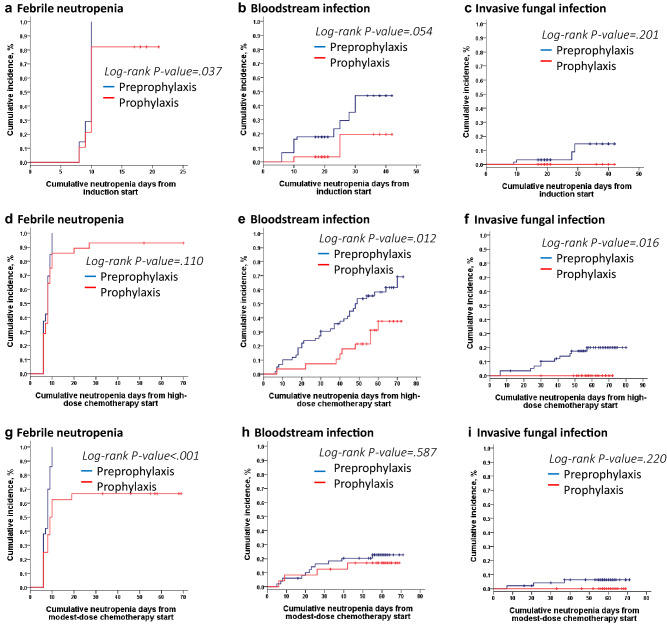
Cumulative incidence of febrile neutropenia, bloodstream infection, invasive fungal infection during induction, high-dose, and modest-dose chemotherapy between the preprophylaxis and prophylaxis period are shown.

There were 87 episodes of BSI and 17 episodes of IFI reported during the preprophylaxis period (see Supplemental Table S3 online). In the prophylaxis period, 16 episodes of BSI and no IFI occurred during chemotherapy. *K. pneumoniae* (28 isolates) was the leading microorganism of BSI and *Aspergillus* spp. (11 isolates) of IFI in the preprophylaxis period, whereas *E. coli* was isolated most commonly (7 isolates) in the prophylaxis period. All episodes of IFI occurred during periods of neutropenia while 3 episodes of BSI occurred without neutropenia during the preprophylaxis period and one during the prophylaxis period. In the prophylaxis period, the reduction in the rates of BSI in patients with AML who were receiving induction or HD chemotherapy and IFI in those receiving HD chemotherapy reached a statistically significant level (Table 2). Patients receiving antimicrobial prophylaxis during HD chemotherapy had a lower cumulative incidence of BSI and IFI in Kaplan–Meier analysis (Fig. 1). The reduction of BSI during the prophylaxis period was also remarkable as using HLM analysis after adjustment for covariates (see Supplementary Table S4 online).

**Table 2:** The rates of BSI, IFI or FN are presenting as the proportion of the total number of BSI, IFI or FN in the course of chemotherapy between the preprophylaxis and prophylaxis period.

	Preprophylaxis period (N = 62)	Prophylaxis period (N = 28)	*P*-value
**Gram (−) BSI (episodes/chemotherapy courses, %)**			
Induction	9/83 (10)	1/36 (3)	0.145
Post-remission			
HD therapy	40/226 (17)	8/116 (7)	0.006
MD therapy	13/193 (7)	4/95 (4)	0.392
Total	62/502 (12)	13/247 (5)	0.002
**Gram (+) BSI**			
Induction	9/93 (10)	0/26 (0)	0.098
Post-remission			
HD therapy	15/256 (6)	2/86 (2)	0.180
MD therapy	2/212 (1)	0/76 (0)	0.395
Total	26/561 (5)	2/188 (1)	0.024
**IFI**			
Induction	3/83 (4)	0/36	0.247
Post-remission			
HD therapy	11/226 (5)	0/116	0.015
MD therapy	3/193 (2)	0/95	0.221
Total	17/502 (4)	0/247	0.003
**FN**			
Induction	82/83 (99)	28/36 (78)	< 0.001
Post-remission			
HD therapy	213/226 (94)	74/116 (64)	< 0.001
MD therapy	112/193 (58)	26/95 (27)	< 0.001
**Total death**	27/62 (44)	6/28 (21)	
Disease related	13/62 (21)	4/28 (14)	
BSI related	7/62 (11)	1/28 (4)	
IFI related	6/62 (10)	0	
Others (HF)	1(2)	1(4)	
Relapse	12/62 (19)	5/28 (18)	
HSCT	6/62 (10)	3/28 (11)	

BSI, bloodstream bacterial infection; FN, febrile neutropenia; HD, high-dose; HF, heart failure; HSCT, hematopoietic stem cell transplantation; IFI, invasive fungal infection; MD, modest-dose; N, number.

The frequencies of FN in induction, HD, or MD chemotherapy were reduced significantly during the prophylaxis period (Table 2), and HLM analysis (Supplementary Table S4) showed similar results. However, a lower cumulative incidence of FN was only in induction or MD chemotherapy during the prophylaxis period under Kaplan–Meier analysis (Fig. 1). Thirteen patients with AML died of infection during the preprophylaxis period (7 of BSIs and 6 of IFIs). In contrast, only one patient with AML, M7 subtype died of *P. aeruginosa* BSI with typhilitis after his fourth course of HD chemotherapy during the prophylaxis period.

**Figure 2:**
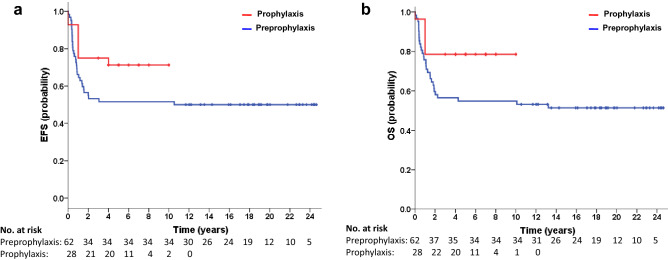
Outcome according to treatment arm. (**a**) Probability of event-free survival (EFS). (**b**) Probability of overall survival (OS) are shown.

At the time of last follow-up (September 30, 2020), the 5-year EFS rate was 51.6% [39.3% to 63.9%] and the 5-year OS rate was 54.8% [42.5 to 67.1%] for 62 patients during the preprophylaxis period; for 28 patients during the prophylaxis period, the rate were 70.6 [53.4% to 87.8%] and 78.6% [63.3% to 93.9%] (Fig. 2).

### Effect of prophylaxis on antimicrobials exposure and susceptibility

**Figure 3:**
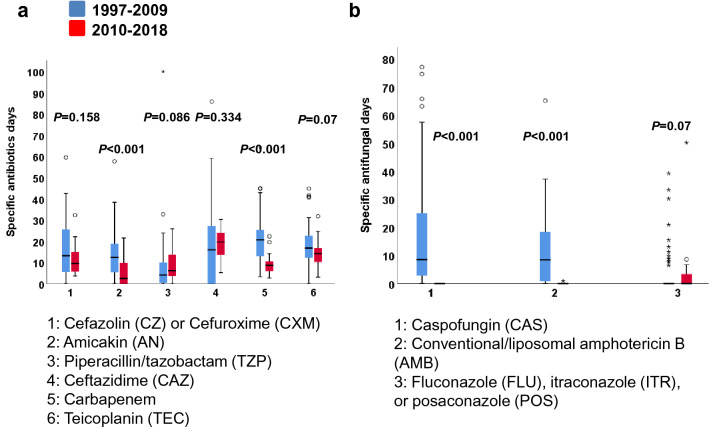
Data of specific antimicrobial days are shown in a box plot; each box plot illustrates the upper and lower quartile (box), median (line inside box), adjacent values (whiskers), and outliers (open circles). Patients receiving antimicrobial prophylaxis had less exposure to carbapenem, amikacin, amphotericin B, or caspofungin when compared with those receiving no prophylaxis (*P* < 0.001*, P* < 0.001*, P* < 0.001, and *P* < 0.001, respectively).

Ciprofloxacin, vancomycin, and voriconazole exposure was significantly greater during prophylaxis period than preprophylaxis period. Antimicrobial exposure was presenting as specific antimicrobial days. There was a concomitant reduction in exposure to carbapenem, amikacin, conventional/liposomal amphotericin B, or caspofungin (*P* < 0.001 for all comparisons; Fig. 3).

**Figure 4:**
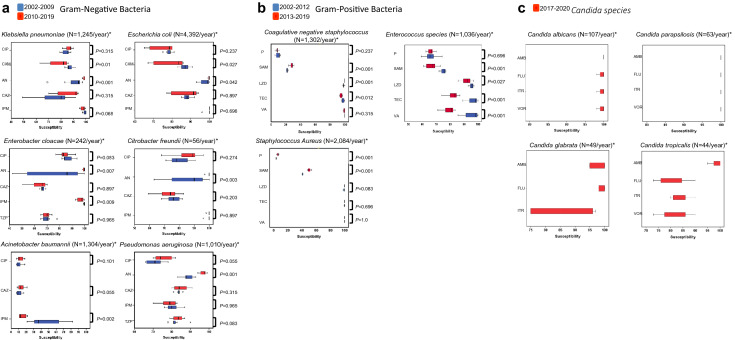
The antibiotic susceptibility of 6 GNB (**a**) and 3 GPB (**b**) between the preprophylaxis and prophylaxis period are shown. Data of specific antimicrobial days are shown in a box plot; each box plot illustrates the upper and lower quartile (box), median (line inside box), and adjacent values (whiskers). (**a**) During the prophylaxis period, cefuroxime (CXM) susceptibility of *E. coli* or *K. pneumonia* were reduced significantly (*P* = 0.027*, P* = 0.01*,* respectively); imipenem (IPM) susceptibility of *E. cloacae* or *A. baumannii* were also reduced (*P* = 0.009, and *P* = 0.002, respectively); a concomitant rising of amikacin (AN) susceptibility of *E. coli*, *K. pneumoniae*, *E. cloacae*, *P. aeruginosa*, *C. freundii* was found (*P* = 0.042*, P* = 0.001*, P* = 0.007, *P* = 0.003, and *P* = 0.001, respectively). (**b**) The ampicillin/sulbactam (SAM), linezolid (LZD), teicoplanin (TEC), or vancomycin (VA) susceptibility of *Enterococcus* species had a significant reduction during the prophylaxis period. The antifungal susceptibility of *Candida species* is shown in (**c**) from 2017 to 2020. *, values are presenting as the median number of infection episodes from any site of body per year from 2002 to 2019 for bacteria and from 2017 to 2020 for *Candida species*. AMB, amphotericin B; CAZ, ceftazidime; CIP, ciprofloxacin; FLU, fluconazole; ITR, itraconazole; P, penicillin; TZP, piperacillin/tazobactam; VOR, voriconazole.

The antibiotic susceptibility of the most common six GNB and three GPB at the study institution from 2002 to 2019 is shown in Fig. 4. The cefuroxime susceptibility of *E. coli* and *K. pneumonia* as well as imipenem susceptibility of *E. cloacae* and *A. baumannii* were reduced significantly during the prophylaxis period. Moreover, there was a concomitant rising of amikacin susceptibility of *E. coli*, *K. pneumoniae*, *E. cloacae*, *P. aeruginosa*, *C. freundii* during the prophylaxis period when compared with the preprophylaxis period. In GPB, the ampicillin/sulbactam, linezolid, teicoplanin, and vancomycin susceptibility of *Enterococcus* spp. had a significant reduction during the prophylaxis period.

### Adverse events

With regard to vancomycin, ciprofloxacin, and voriconazole toxicity, 6 patients (24%) had hypersensitivity reactions, red man syndrome, during initial vancomycin prophylaxis. The reactions were well prevented by slowing the injection rates and antihistamine premedication during the following prophylaxis courses. There were 12 episodes (6%) of elevated transaminase levels, 10 (5%) of grade 1 and 2 (1%) of grade 2, reported in 8 patients while no renal toxicity or hypokalemia were observed during antimicrobial prophylaxis.

## Discussion

There are several findings from the current study. First, prophylaxis treatment effectively decreased the occurrence of BSIs in pediatric AML patients receiving induction chemotherapy, and both BSIs and IFIs in post-remission HD chemotherapy. Second, prophylaxis treatment significantly reduced the episodes of FN as well as death related to life threatening infections during chemotherapy. Third, exposure to antibiotics, especially amikacin and carbapenem, and antifungal agents such as Caspofungin and amphotericin-B during the prophylaxis period were significantly lower than those for the treatment of any infections during the preprophylaxis period. Fourth, we demonstrated the degree of amikacin susceptibility against *E. coli*, *K. pneumoniae*, *E. cloacae*, *P. aeruginosa*, and *C. freundii* was improved during the prophylaxis period. Furthermore, although the carbapenem exposure declined during the prophylaxis period, the resistance to imipenem among *E. cloacae* and *A. baumannii* were more common. The rate of multidrug-resistant Enterococci was increased remarkably during the prophylaxis period. Finally, in this comparative historical study, the overall outcome in pediatric AML patients was improved significantly during the prophylaxis period in comparison to the preprophylaxis period.

The advancement of supportive care measures have, doubtlessly, contributed significantly to the improvement in outcomes for children with AML patients. Studies conducted by the AML-BFM group and the St. Jude AML trials showed that decreased treatment-related mortality in AML patients improved over successive clinical trials in the 1990s and 2000s^3,18^. Infectious complications remain a major cause of morbidity and mortality for pediatric AML patients who are at particular risk of Viridians Group Streptococci (VGS), GNB, and IFI^19^. A high incidence of VGS and GNB infections in pediatric AML^6,19^ administration of antibiotics prophylaxis was reasonable to reduce the rates of BSI during intensive treatment^5,20^. In the study institution, several infection control practices other than antimicrobials prophylaxis have been added over time to try to reduce the incidence of infection. First, the pediatric intensive care unit was renovated in 2008 and the children’s oncology ward in 2013 since the hospital building was more than 30 years old. Second, a training program for oncology clinical nurse specialists has been held every 2 years since 2007. Third, a low-bacterial diet was started to provide for children with cancer receiving chemotherapy during hospitalization. These practices might be contributed to the reduction infection rates of children with cancer undergo chemotherapy.

Currently, there are no comparative studies of prophylaxis with vancomycin in children with AML who underwent chemotherapy. Kurt et al. reported that prophylaxis with vancomycin-containing regimens markedly reduced the odds of VGS sepsis by 99% relative to no prophylaxis^20^. The present report demonstrated the cumulative incidence of gram-positive BSI was 5% of chemotherapy courses in pediatric AML without vancomycin prophylaxis compared to 1% for patients with prophylaxis, and the reduction in rates of GPB BSI was significant in the prophylaxis period.

Regarding the adverse effects of vancomycin during the period of vancomycin prophylaxis, only minimal elevated transaminase levels and no nephrotoxicity were found. The results of minimal hepatic and renal toxicity during vancomycin prophylaxis in pediatric AML were similar to one recent report^21^. Compared with the therapeutic dose of vancomycin, the rate of nephrotoxocity was 8–20% as vancomycin troughs was maintained between 10 and 15 mg/l^22^. We believe that routine therapeutic vancomycin level monitoring is not necessary during the use of vancomycin prophylaxis. Although the susceptibility of vancomycin to two most common GPB, coagulase-negative *staphylococcus* and *staphylococcus aureus*, were no different during the prophylaxis period, the susceptibility of many classes of antibiotics including vancomycin, tecoplanin, linezolid, and ampicillin/sulbactam to *Enterococcus* species were significantly reduced during the prophylaxis period in the study institution. The trend of increasing the proportion of Vancomycin-resistant *Enterococcus* was noted so we start to omit prophylaxis with vancomycin for AML patients receiving MD chemotherapy in order to reduce vancomycin exposure in patients with low infection risk. Further research should aim to compare the efficacy, toxicity, and antibiotics susceptibility of vancomycin-based prophylactic regimen and alternative regimens in independent cohorts.

Development of antimicrobial resistance has increased dramatically over the past few years and has become a great public health problem worldwide. GNB such as *E. coli* and *K. pneumoniae* have displayed increasing resistance to second-, third-, or fourth-generation cephalosporins for neonatal and infant sepsis, ranging from 23 to 51%, in certain developing countries^23,24^. Moreover, the epidemiology of carbapenem-resistant enterobacteriaceae had increased from 1 to 12% in US hospitals^25^ with a 50% associated mortality rate^26^ between 2000 and 2010. Therefore, we put forward a report on changes in the antibiotic susceptibility of bacteria at the study institution and analyze the impact of antimicrobial prophylaxis strategies on antibiotic susceptibility of bacteria. This has not been previously reported in detail in pediatric AML patients under antimicrobial prophylaxis^8,9^. In the present study, the number of days used for amikacin or carbapenam was significantly reduced in children with AML during the prophylaxis period. Several possible reasons are as follows: first, empiric or therapeutic amikacin was used less frequently due to the concern of its nephrotoxicity. Second, episodes of FN or BSI were occurred less frequently, thus reducing the number of days used for carbapenem. Although the number of days used for carbapenem was reduced during the prophylaxis period, some bacteria, such as *E. cloacae* or *A. baumannii*, observed were less sensitive to carbapenem at study institution. This may be due to the fact that the study institution is one general children’s hospital that treats children with different kinds of acute or chronic illnesses. Late-line antibiotics were used frequently for the treatment of severe bacterial infections. Therefore, the sensitivity of bacteria to carbapenem has gradually reduced in recent years. We have tried to maintain carbapenam resistance by administering other antimicrobials (eg. cephalosporin and aminoglycoside) when patients are found to have GNB BSI and reserving carbapenam as a second-line antibiotic.

Among 16 episodes of BSI during the prophylaxis period, 13 (81%) were breakthrough BSI (bBSI). Susceptibility of 13 bBSI to cefuroxime, ceftazidime, amikacin, piperacillin/tazobactam, imipenem, and ciprofloxacin were 43, 50, 93, 64, 93%, and 0%, respectively, in the present study. Cefuroxime and amikacin were administered empirically for FN during ciprofloxacin prophylaxis because their susceptibility rate to major GNB was more than 90% at the study institution. For patients with renal insufficiency, amikacin was not administered and piperacillin/tazobactam was used to preserve their renal function. Carbapenam was reserved for patients with bBSI only in order to have effective therapy for GNB and maintaining its resistance.

Since ciprofloxacin has been used as a prophylaxis in neutropenic patients, the susceptibility of ciprofloxacin to the most common 6 GNB at the study institution from 2002 to 2019 was investigated. A part of the antimicrobial susceptibility result has been reported previously^5^ and this study included additional patients with longer follow-up. Our previous 3-year study^5^ reported the susceptibility of ciprofloxacin to *E. coli*, *K. pneumoniae*, *P. aeruginosa*, and *Serratia marcescens* significantly increased during the prophylaxis period, whereas no significant changes in susceptibility to ciprofloxacin between the preprophylaxis and prophylaxis periods in the present study. Ciprofloxacin was selected continuously as a prophylactic agent because of its susceptibility rate to major GNB was around 80–90% at the study institution. However, one global surveillance study demonstrated that fluoroquinolone resistance rates increased in the past years in almost all bacterial species^27^. We have proposed several strategies to preserve ciprofloxacin susceptibility: first, administering other antimicrobials (eg. cephalosporin) when patients have GNB infections and reserving ciprofloxacin as a second-line antibiotic; second, administering G-CSF in patients with profound neutropenia after intensive chemotherapy in order to reduce the neutropenic period, therefore, ciprofloxacin exposure might be reduced; third, antimicrobial prophylaxis with cefepime instead of ciprofloxacin for neutropenic patients requiring secondary prophylaxis.

In conclusion, the current study provides new information by comparing prophylaxis with antimicrobials in children with AML without prophylaxis. All patients were treated according to a single protocol at a single institution and were provided with standard supportive care. For patients undergoing intensive chemotherapy, antimicrobials prophylaxis was found to significantly reduce the rate of BSIs and IFIs. Prophylaxis also reduced the episodes of FN and the mortality caused by severe infections. The exposure of amikacin, carbapenem, caspofungin, and amphotericin-B were significantly reduced and overall survival was improved significantly during the prophylaxis period. These results support the importance of antibiotics and antifungal prophylaxis to prevent treatment related morbidities, especially BSIs and IFIs, for children with AML.

## Supplementary Information

Supplementary Information.

## Data Availability

The data are available on request from the authors.
