# Supplementary material for: Effectiveness and antimicrobial susceptibility profiles during primary antimicrobial prophylaxis for pediatric acute myeloid leukemia
Source: Sci Rep. 2021 Oct 27;11:21142. doi: 10.1038/s41598-021-00725-5 (PMC8551163; doi:10.1038/s41598-021-00725-5)
Supplement: Supplementary file 1 — Supplementary Information. [file 41598_2021_725_MOESM1_ESM.pdf]

Title: Effectiveness and antimicrobial susceptibility profiles during primary antimicrobial prophylaxis for pediatric acute myeloid leukemia

Authors: Ting-Chi Yeh, Jen-Yin Hou, Ting-Huan Huang, Chien-Hung Lu, Fang-Ju Sun, Hsiu-Mei Huang, Hsi-Che Liu

SUPPLEMENTAL FIGURE S1. Acute Myeloid Leukemia 97A treatment schema

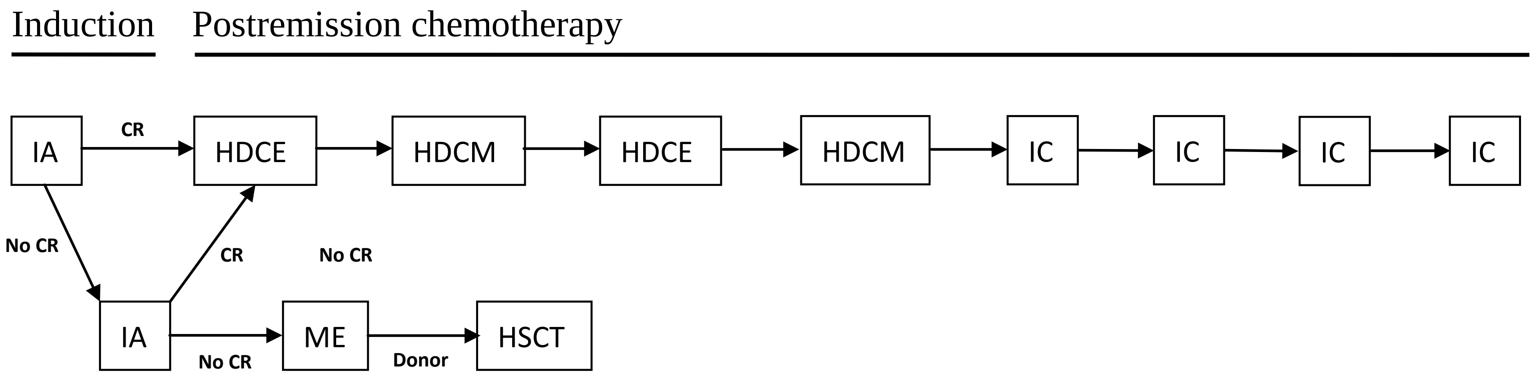

IA: Idarubicin (9 mg/m^2/d for 3d) and Ara-C (100 mg/m^2/d for 7d), IT MTX

ME: Mitoxantrone (8 mg/m^2/d for 5d) and Etoposide (100 mg/m^2/d for 5d)

HDC: Ara-C (1 g/m^2/12 h on d 1 to 4); E: Etoposide (100 mg/m^2/d for 5d); M: Mitoxantrone (10 mg/m^2/d on d 2 to 5)

IC: Idarubicin (9 mg/m^2/d on d1) and Ara-C (200 mg/m^2/d for 5 d), IT MTX

IT: MTX 6-12mg age dependent, day 1

Abbreviations: CR, complete remission; HSCT, hematopoietic stem cell transplantation; IT, intrathecal chemotherapy

SUPPLEMENTAL FIGURE S2. Patient flow chart during the study

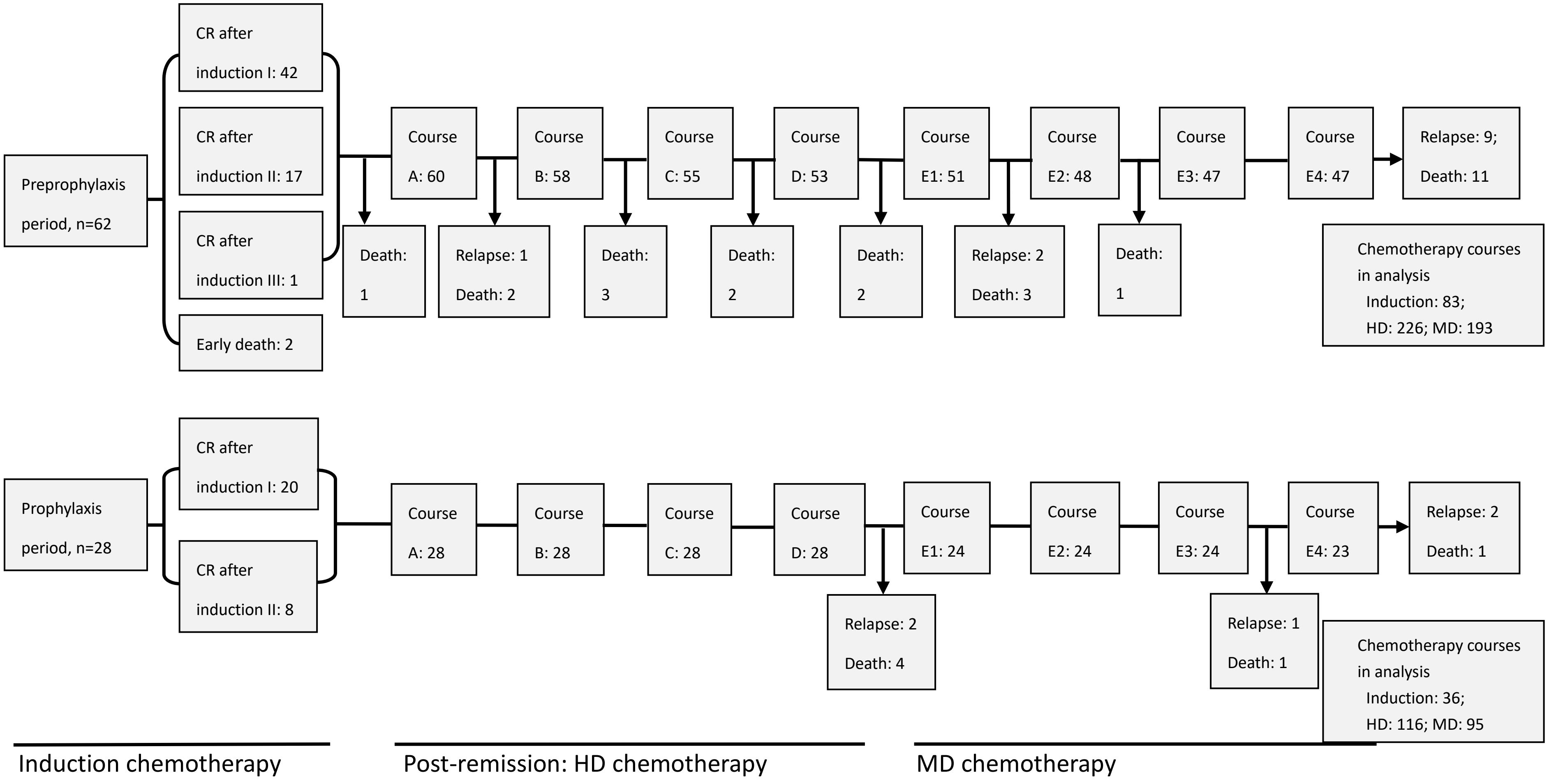

Abbreviations: CR, complete remission; HD, high-dose; MD, modest-dose

SUPPLEMENTAL TABLE S3. Correspondence between the occurrence of BSI and IFI and the course of chemotherapy

Course of chemotherapy		Preprophylaxis period	Prophylaxis period
Induction I	GP	MRCNS (5); VGS (1)	VGS (1)*
	GN	*K. pneumoniae* (2); *E. coli* (1); *P. aeruginosa* (1); *S. maltophilia* (1)	
	IFI	*Aspergillus flavus*, *Aspergillus* species, *Penicillum* species	
Induction II/III	GP	MRCNS (2)	
	GN	*K. pneumonia*/*E. cloacae* (1)**; *A. baumannii* (1); *E. cloacae* (1); *S. maltophilia* (1)	*K. pneumoniae* (1)
	IFI	*Candida albicans*	
Postremission HD Ara-C, course A	GN	*K. pneumoniae* (3); *K. pneumonia*/*E. coli* (1)**; *A. baumannii* (1); *A. calcoaceticus* (1)	*E. coli* (1)
	IFI	*Aspergillus* species, *Candida* species	
Course B	GP	VGS (1); MRCNS (4); VGS/MRCNS (1)**	
	GN	*K. pneumoniae* (3); *P. aeruginosa* (1); *A. baumannii* (1); *C. freundii* (1); *S. marcescens* (1)	*E. coli*/*P. aeruginosa* (1)**; *P. aeruginosa* (1)
	IFI	*Aspergillus* species, *Candida tropicalis/Aspergillus* species**, *Candida tropicalis*	
Course C	GP	VGS (1); MRCNS (1); VRE (1); *Corynebacterium* (1)	
	GN	*K. pneumoniae* (5); *E. coli* (2); *C. freundii* (1); *E. cloacae* (1)	*E. coli* (2); *C. freundii* (1)
	IFI	*Aspergillus* species(3)	
Course D	GP	MRCNS (2); VGS (1); *S. pneumoniae* (1); *Corynebacterium* (1)	VGS (1)*; VRE (1)
	GN	*K. pneumoniae* (7); *E. coli* (7); *K. oxytoca* (1); *P. aeruginosa* (1); *A. baumannii* (1); *E. cloacae* (1)	*E. coli* (2)
	IFI	*Candida* species(2), *Aspergillus* species	
Postremission MD	GP	*Corynebacterium* (1)	

Ara-C, course E1	GN	*K. pneumoniae* (2); *P. aeruginosa* (1); *E. cloacae* (1)	*A. baumannii* (1); *E. coli* (1)
	IFI	*Aspergillus* species	
Course E2	GN	*K. pneumonia*/*C. freundii* (1)**; *E. coli* (1); *E. cloacae* (2)	*K. pneumoniae* (1)
	IFI	*Aspergillus* species	
Course E3	GN	*K. pneumoniae* (3); *E. cloacae* (1)	*K. pneumoniae* (1)
	IFI	*Blastoschizomyces Capitatus*	
Course E4	GP	MRCNS (1)	
	GN	*E. cloacae* (1)	

*: VGS occurrence without vancomycin prophylaxis

**: Two microorganisms were isolated concomitantly

Abbreviations: *A. baumannii*, *Acinetobacter baumannii*; *A. calcoaceticus*, *Acinetobacter calcoaceticus*; *C. freundii*, *Citrobacter freundii*; *E. cloacae*, *Enterobacter cloacae*; *E. coli*, *Escherichia coli*; GN, Gram-negative bacteria; GP, Gram-positive bacteria; HD, high-dose; IFI, invasive fungal infection; *K. oxytoca*, *Klebsiella oxytoca*; *K. pneumonia*, *Klebsiella pneumoniae*; MD, modest-dose; MRCNS, Methicillin-resistant coagulase-negative *staphylococcus*; *P. aeruginosa*, *Pseudomonas aeruginosa*; *S. marcescens*, *Serratia marcescens*; *S. maltophila*, *Stenotrophomonas maltophila*; *S. pneumoniae*, *Streptococcus pneumoniae*; VRE, Vancomycin-resistant *Enterococcus*; VGS, Viridans group streptococci

SUPPLEMENTAL TABLE 4. Hierarchical linear modeling analysis for effectiveness of primary prophylaxis

a Febrile neutropenia

	Induction chemotherapy		High-dose chemotherapy		Modest-dose chemotherapy		Total	
	OR (95% CI)	*P*-value	OR (95% CI)	*P*-value	OR (95% CI)	*P*-value	OR (95% CI)	*P*-value
Prophylaxis	0.140 (0.028, 0.706)	0.018**	0.105 (0.045, 0.245)	<0.001**	0.010 (0.003, 0.029)	<0.001**	0.03 (0.01, 0.06)	<0.001**
Age*								
Age ≥10years	1.351 (0.187, 9.755)	0.763	1.593 (0.497, 5.108)	0.432	2.367 (0.523, 10.714)	0.262	1.82 (0.67, 4.95)	0.238
Age 1-10years	2.386 (0.291, 19.567)	0.415	1.712 (0.555, 5.274)	0.348	1.531 (0.367, 6.383)	0.558	1.84 (0.71, 4.76)	0.211
Mucositis	1.205 (0.213, 6.830)	0.832	0.682 (0.261, 1.779)	0.433	1.250 (0.466, 3.351)	0.656	1.16 (0.64, 2.12)	0.620
Parenteral neutrition use	0.566 (0.111, 2.890)	0.491	1.504 (0.493, 4.586)	0.472	2.215 (0.778, 6.309)	0.136	1.47 (0.72, 2.98)	0.286
Neutropenia days	1.215 (0.603, 2.447)	0.583	0.992 (0.867, 1.135)	0.905	1.068 (0.916, 1.246)	0.400	1.09 (0.99, 1.19)	0.068

b Bloodstream infection

	Induction chemotherapy		High-dose chemotherapy		Modest-dose chemotherapy		Total	
	OR (95% CI)	*P*-value	OR (95% CI)	*P*-value	OR (95% CI)	*P*-value	OR (95% CI)	*P*-value
Prophylaxis	0.292 (0.067, 1.267)	0.099	0.292 (0.133, 0.640)	0.002**	0.540 (0.135, 2.167)	0.383	0.35 (0.18, 0.68)	0.002**
Age*								
Age ≥10years	0.288 (0.068, 1.232)	0.092	1.530 (0.598, 3.913)	0.374	0.959 (0.171, 5.395)	0.962	1.08 (0.52, 2.25)	0.830
Age 1-10years	0.439 (0.127, 1.514)	0.190	1.193 (0.487, 2.926)	0.699	0.977 (0.211, 4.519)	0.976	0.89 (0.45, 1.77)	0.749
Mucositis	1.338 (0.397, 4.502)	0.636	0.634 (0.277, 1.448)	0.278	0.983 (0.284, 3.399)	0.978	1.07 (0.63, 1.79)	0.811
Parenteral neutrition use	0.495 (0.121, 2.025)	0.325	1.803 (0.715, 4.543)	0.211	1.407 (0.430, 4.611)	0.571	1.20 (0.67, 2.17)	0.540
Neutropenia days	1.153 (0.753, 1.765)	0.510	0.963 (0.860, 1.079)	0.516	1.098 (0.907, 1.329)	0.335	1.04 (0.96, 1.12)	0.311

c Invasive fungal infection

	Induction chemotherapy		High-dose chemotherapy		Modest-dose chemotherapy		Total	
	OR (95% CI)	*P*-value	OR (95% CI)	*P*-value	OR (95% CI)	*P*-value	OR (95% CI)	*P*-value
Prophylaxis	0.458 (0.050, 4.176)	0.486	0.367 (0.107, 1.256)	0.110	0.829 (0.187, 3.674)	0.804	0.52 (0.21, 1.28)	0.154
Age*								
Age ≥10years	0.943 (0.085, 10.485)	0.961	4.051 (0.765, 21.449)	0.100	0.760 (0.111, 5.182)	0.778	1.90 (0.66, 5.52)	0.237
Age 1-10years	0.937 (0.104, 8.437)	0.953	1.512 (0.272, 8.401)	0.636	0.972 (0.183, 5.173)	0.973	1.22 (0.43, 3.49)	0.706
Mucositis	1.407 (0.214, 9.247)	0.720	1.102 (0.308, 3.939)	0.881	1.658 (0.397, 6.931)	0.487	1.44 (0.64, 3.22)	0.377
Parenteral neutrition use	0.368 (0.031, 4.361)	0.424	1.260 (0.299, 5.318)	0.752	0.724 (0.122, 4.290)	0.721	0.78 (0.29, 2.09)	0.627
Neutropenia days	0.877 (0.442, 1.742)	0.705	0.896 (0.739, 1.085)	0.259	0.947 (0.743, 1.208)	0.661	0.95 (0.84, 1.08)	0.429

*reference group, age<1year

** $P < 0.05$

OR, odds ratio calculated by hierarchical linear modeling; CI, confidence interval
